# Evaluation of the relationship between resilience and empathy in medical professionals using structural equation modeling

**DOI:** 10.3389/fpsyg.2026.1787763

**Published:** 2026-05-08

**Authors:** Heberto R. Priego-Alvarez, Juan A. Córdova-Hernández, María I. Avalos-García, José Gamarra-Moncayo, Víctor P. Díaz-Narváez

**Affiliations:** 1 División Académica de Ciencias de la Salud, Universidad Juárez Autónoma de Tabasco, Villahermosa, Mexico; 2 Facultad de Medicina, Universidad Católica Santo Toribio de Mogrovejo, Chiclayo, Peru; 3 Departamento de Investigaciones, Facultad de Odontología, Universidad Andres Bello, Santiago, Chile

**Keywords:** cross-sectional study, empathy, physicians, psychometrics, residents, resilience, structural equations modeling

## Abstract

**Background:**

Research has demonstrated that resilience serves as a shield against negative experiences. Some studies have explored how resilience helps maintain physicians’ empathetic behavior toward patients. However, these studies have not investigated how specific dimensions of resilience protect different aspects of empathy.

**Objective:**

The aim of this study is to explore whether resilience can predict empathetic expression in Mexican physicians by analyzing how each dimension of resilience predicts each dimension of empathy.

**Methods:**

The study population (*N* = 1,067) includes specialist and resident physicians who provide care at five hospitals of the Instituto Mexicano del Seguro Social (IMSS) Bienestar in Villahermosa, Mexico. The IMSS Bienestar is the country’s public healthcare institution. The sample (*n* = 314, or 29.43% of the total population) comprises both male and female participants and is non-random, as participation is voluntary. Resilience was assessed using the Trait Scale, while empathy was measured with the Jefferson Empathy Scale for Health Professionals. Descriptive statistics, including mean, standard deviation, skewness, and kurtosis, were calculated for each dimension of both constructs. Psychometric analyses were conducted, including confirmatory factor analysis, model fit testing, and invariance testing. Structural equation modeling was used to assess the predictive relationship between each dimension of resilience and each dimension of empathy.

**Results:**

The results revealed that both ecological and engineering resilience dimensions are positively associated with all dimensions of empathy. However, adaptive resilience showed a negative correlation with Compassionate Care and the “Walking in the patient’s shoes” dimension of empathy.

**Conclusion:**

Each dimension of resilience can predict the corresponding dimensions of empathy. This prediction helps identify the specific traits within each resilience dimension that are essential for fostering a protective response to negative events. It also highlights which traits, when lacking, may potentially reduce the overall expression of empathy among the physicians studied.

## 1 Introduction

Empathy plays a crucial role in providing humanized patient care (Busch et al., 2019). As such, one could argue that humanized care cannot be achieved without physicians who consistently demonstrate empathy toward their patients (Morales-Garrido et al., 2025). A lack of empathy often results from deficiencies in one or more of its core dimensions. Empathy functions as a system, and in such systems, any deficiency or failure in one component inevitably impacts the overall functioning of the system (Díaz-Narváez et al., 2024).

This attribute facilitates the development of an intersubjective relationship between physician and patient, which is mutually beneficial (Busch et al., 2019; Cameron and Inzlicht, 2020; Derksen et al., 2013; Díaz-Narváez et al., 2025a). The literature extensively documents these benefits for both patients and physicians (Cameron and Inzlicht, 2020; Derksen et al., 2013; Díaz-Narváez et al., 2025a).

The key dimensions of empathy in relation to patients include Compassionate Care (CC), Patient Perspective Adoption (PA), and Walking in the Patient’s Shoes (WIPS) (Díaz-Narváez et al., 2025a; Díaz-Narváez et al., 2025b). Compassionate Care is generally considered an emotional component (Weisz and Cikara, 2021), while the other two are categorized as cognitive components (Coundouris et al., 2020). There is a biological connection between these components; the emotional aspect is typically linked to the activity of the limbic system (Healey and Grossman, 2018), whereas the cognitive aspect is associated with the orbitofrontal cortex. Like any system, if one of its essential elements is compromised, the overall system fails to function properly. In extreme cases, a significant lack or absence of compassion can lead to serious consequences for empathetic functioning, such as in conditions like narcissism, psychopathy, or malignant personality disorder (Shukla and Upadhyay, 2025).

In the case of resilience, which serves a protective role against negative events, its deficiency might partly explain the decline in empathy observed in medical students or the erosion of empathy (Blanco et al., 2020) in physicians, especially those working in hospitals and public healthcare centers (Díaz-Narváez et al., 2024; Díaz-Narváez et al., 2025a; Díaz-Narváez et al., 2025b). The level of empathy a physician develops can be influenced by various factors, including family dynamics (Ulloque et al., 2023), personality (Salgado et al., 2020), stress (which can lead to anxiety and depression) (Jamil et al., 2025), the academic environment during training (Dyrbye et al., 2021), self-efficacy (Pérez-Fuentes et al., 2019), and resilience (Waddimba et al., 2021), among others (Díaz-Narváez et al., 2025b). However, research specifically examining how each dimension of resilience relates to each dimension of empathy is still limited.

Resilience has been identified as an attribute that helps medical personnel counteract the negative effects of challenging events on their professional performance (Díaz-Narváez et al., 2025a; Ulloque et al., 2023) and serves as a protective factor against any reduction in their ability to express empathy toward patients (Acosta-Martínez et al., 2024). Despite its importance, literature has yet to fully clarify how resilience influences empathy, as the theory of resilience is still evolving (Díaz-Narváez et al., 2025b). Consequently, further empirical research is needed to provide insights that will contribute to the development and refinement of this theory.

**Figure 1:**
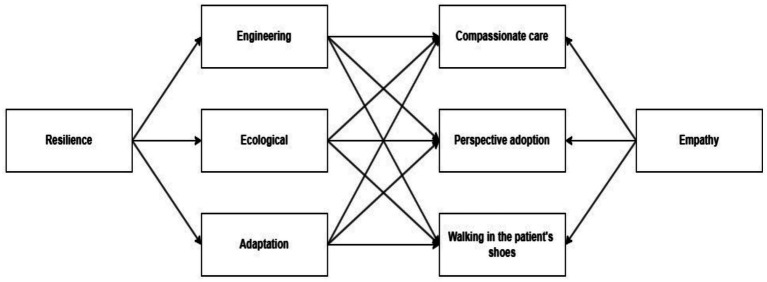
Theoretical model of resilience as a predictor of empathy.

Several authors have suggested that resilience acts as a protective attribute, reducing or nullifying the impact of negative events or disturbances, protecting psychological stability, mental health, and the empathic capacity achieved by the medical professional, it is possible to conceptualize resilience as an independent variable (Acosta-Martínez et al., 2024; Díaz-Narváez et al., 2025a; Díaz-Narváez et al., 2025b; Ulloque et al., 2023) with a relationship between these two attributes. In any case, there is still discussion regarding directionality, and it is a problem that has not yet been resolved. Although the relationship between resilience and empathy may be bidirectional, the present study adopts a unidirectional model in which resilience predicts empathy, based on theoretical perspectives that conceptualize resilience as a regulatory and adaptive capacity that may facilitate socio-emotional functioning (Figure 1).

Therefore, the aim of this study is to assess whether the protective role of resilience in empathy can predict the empathetic capacity of Mexican physicians. This will be based on analyzing how each dimension of resilience predicts the corresponding dimensions of empathy, with resilience being considered as an independent variable in this theoretical framework.

## 2 Methods

### 2.1 Design

Observational and cross-sectional.

### 2.2 Participants

The population consisted of 1,067 medical professionals (697 attending physicians and 370 resident physicians), employed by the IMMS Bienestar who provided direct patient care in five hospitals in the city of Villahermosa, Tabasco, Mexico. The sample consisted of 314 participants (54.5% women), including 110 attending physicians and 204 medical residents, aged between 21 and 73 years (*M* = 33.77; SD = 8.52). This sample represents 29.43% of the total population. Both subgroups were combined for analysis, as the focus was on understanding the overall empathetic behavior of medical professionals within the context of several public hospitals in the state of Tabasco, Mexico, rather than distinguishing between the two groups. Given that participation in the study was voluntary, the sampling method used can be considered convenience sampling.

Inclusion and exclusion criteria. Inclusion: All physicians who voluntarily agreed to complete the questionnaires. Exclusion: Physicians who completed the questionnaires but did so incompletely or who did not sign the informed consent form.

### 2.3 Instruments

Jefferson Scale of Empathy-Health Professions Students (JSE-HPS) (Hojat et al., 2023). This instrument was used under license from Asano-Gonnella Center for Research in Medical Education and Health Care Thomas Jefferson University (Order ID: 11029). This scale consists of 20 items that measure levels of empathy toward patients in health sciences students from any specialty. Items are rated on a 7-point response scale ranging from 1 (Strongly Disagree) to 7 (Strongly Agree). The scale measures three dimensions: Compassionate Care (CC) (Items 1, 7, 8, 11, 12, 14, 18, 19), Perspective Adoption (PA) (Items 2, 4, 5, 9, 10, 13, 15, 16, 17, 20) and Walking in the Patient’s Shoes (WIPS) (Items 3 and 6).

The scale has demonstrated adequate internal consistency (*α* = 0.78–0.92) and appropriate correlations with other psychological variables (Hojat et al., 2023), and intercultural stability (Ulloque et al., 2023). This instrument is not designed to determine pathological conditions (Díaz-Narváez et al., 2025a; Díaz-Narváez et al., 2024; Ulloque et al., 2023).

Trait Resilience Scale (EEA) (Maltby, 2025). It assesses three facets of resilience: engineering resilience (4 items), ecological resilience (4 items), and adaptive resilience (4 items). The EEA Trait Resilience scale has demonstrated adequate internal consistency and test–retest reliability, a stable cross-cultural factor structure, convergent and construct validity in terms of associations with personality, and a positive contribution to clinical and non-clinical psychological health states (Maltby, 2025; Maltby et al., 2015).

### 2.4 Procedure

The instruments were administered in paper format along with the corresponding informed consent forms in 2025. The instruments were administered by medical graduates from the University of Tabasco, Mexico. Study personnel were previously trained to explain the importance of this research and to answer any questions study participants might have regarding the instruments. The instruments were completed in the rooms designated for clinical meetings at the respective hospitals participating in this study, in a calm environment, except for emergency cases that required the sudden departure of the respondents from the room, in which case the instruments were administered later, but in the same context described above.

### 2.5 Data analysis

Data analysis started with the application of psychometric tests to empathy and resilience scales, as it is essential to verify their functionality to ensure the validity and reliability of the collected data (Kline, 2023).

Descriptive statistics were computed, and confirmatory factor analysis (CFA) was conducted using the Maximum Likelihood Robust (MLR) estimator for the empathy scale, as it includes seven response options treated as continuous data (Rhemtulla et al., 2012). For the resilience scale, the Weighted Least Squares Mean and Variance Adjusted (WLSMV) estimator was applied, since it contains five response options treated as ordinal data (Kline, 2023). Additionally, the fit of the model was assessed using the Comparative Fit Index (CFI) (>0.90), Tucker-Lewis Index (TLI) (>0.90), Root Mean Square Error of Approximation (RMSEA) (<0.08), and Standardized Root Mean Square Residual (SRMR) (<0.08) as indicators of good model fit (Whittaker and Schumacker, 2022).

Reliability was calculated using the omega coefficient, where values greater than 0.70 are considered adequate (Viladrich et al., 2017) Invariance was corroborated according to sex, considering the cut-off points proposed by Chen (2007): ΔCFI (<−0.01) and ΔRMSEA (>0.015), and cut-off points were calculated (Muñiz, 2018) considering five levels for both instruments.

After confirming the proper psychometric functioning of the instruments, the dimensions of resilience were modeled as predictors of the dimensions of empathy using structural equation modeling. To assess the adequacy of the proposed model, the same fit indices used in the CFA were applied. The analyses were conducted using JASP 0.95.2 software and the R programming language within the RStudio environment, utilizing the *lavaan* (0.6–20) and *semTools* (0.5–7) packages.

### 2.6 Ethical considerations

The research was conducted in accordance with the Declaration of Helsinki (World Medical Association, 2013), and before completing the questionnaire, the physicians signed an informed consent form, guaranteeing their voluntary and confidential participation. The study was approved by the Research Ethics Committee of the IMSS Bienestar Regional High-Specialty Hospital de la Mujer, with registration number HRAEM CEI-2025-028.

## 3 Results

Table 1 shows that items 2, 4, and 20 of the empathy scale have indices above the cut-off point for skewness and/or kurtosis (|2|), suggesting the presence of univariate non-normality (Bandalos and Finney, 2019).

**Table 1:** Univariate descriptive statistics for the items of empathy and resilience scales.

Items	*M*	SD	g1	g2	Items	*M*	SD	g1	g2
Empathy scale									
1	3.60	2.34	0.30	−1.46	11	5.49	1.87	−1.08	0.02
2	6.43	0.98	−1.91	3.45	12	5.46	1.93	−1.08	−0.13
3	5.31	1.90	−0.91	−0.40	13	5.76	1.50	−1.24	1.03
4	6.34	1.16	−2.17	5.14	14	5.80	1.81	−1.46	0.91
5	5.66	1.43	−0.96	0.42	15	5.94	1.48	−1.50	1.69
6	5.15	1.85	−0.75	−0.57	16	5.99	1.19	−1.02	0.31
7	5.58	1.83	−1.15	0.15	17	5.67	1.48	−0.94	0.26
8	4.33	2.24	−0.16	−1.43	18	3.04	1.87	0.55	−0.71
9	5.81	1.47	−1.31	1.17	19	5.96	1.63	−1.51	1.20
10	5.85	1.38	−1.22	1.00	20	6.32	1.14	−1.82	2.87
Resilience scale									
1	4.10	0.92	−0.91	0.39	7	4.33	0.75	−1.15	1.84
2	3.92	0.97	−0.64	−0.30	8	4.29	0.80	−1.19	1.76
3	3.99	0.91	−0.78	0.37	9	3.54	1.13	−0.43	−0.47
4	3.93	0.93	−0.65	−0.07	10	3.34	1.16	−0.23	−0.68
5	4.38	0.79	−1.36	2.18	11	3.33	1.16	−0.30	−0.66
6	4.15	0.84	−0.94	0.79	12	3.43	1.19	−0.34	−0.71

M, Mean; SD, Standard deviation; g1, Skewness; g2, Kurtosis.

Table 2 shows that the internal structure of the empathy and resilience scales was valid.

**Table 2:** Confirmatory factor analysis for empathy and resilience scales.

Scale	X2 (gl)	*p*	CFI	TLI	RMSEA [IC 90%]	SRMR
Empathy	263.22 (167)	< 0.001	0.94	0.93	0.04 [0.03–0.05]	0.05
Resilience	165.43 (51)	< 0.001	0.98	0.98	0.08 [0.07–0.09]	0.05

Reliability, assessed using the omega coefficient, was acceptable for both the empathy scale dimensions (caring with compassion = 0.79; perspective adoption = 0.86; and walking in the patient’s shoes = 0.75) and resilience (engineering = 0.92; ecological = 0.93; and adaptation = 0.87).

Table 3 reports that the empathy scale is strictly invariant across gender. However, this invariance could not be demonstrated for resilience.

**Table 3:** Invariance across gender for the empathy scale.

Scale	Level	X2 (gl)	*p*	CFI	ΔCFI	RMSEA	ΔRMSEA	SRMR
Empathy	Configural	514.02 (334)	<0.001	0.895		0.059		0.066
	Metric	531.51 (351)	<0.001	0.895	0	0.057	−0.002	0.073
	Scalar	555.24 (368)	<0.001	0.891	−0.004	0.057	0	0.075
	Strict	566.10 (388)	<0.001	0.896	0.005	0.054	−0.003	0.076

Table 4 presents the results of the estimation of the mean and standard deviation of the respective dimensions of both constructs studied and the values of each construct overall.

**Table 4:** Results of the mean and standard deviation and their respective dimensions in the empathy and resilience constructs.

Dimensions	*n*	M	SD
Compassionate care	314	39.26	9.987
Perspective adoption	314	59.75	8.768
Walking in the patient’s shoes	314	10.46	3.348
Empathy	314	109.48	17.168
Engineering resilience	314	15.94	3.270
Ecological resilience	314	17.15	2.613
Adaptive resilience	314	13.64	3.844
Resilience	314	46.73	7.527

n, sample size; M, Mean; SD, standard deviation.

Table 5 presents cutoff points for the empathy and resilience scales, considering five classification levels.

**Table 5:** Cutoff points for empathy and resilience scales.

PC	CC	PA	WIPS	EMP	ENG	ECO	ADA	RES	Level
99	56	70	14	140	20	20	20	60	Extremely high
90	55	69	13	130	19	19	19	57	
80	48	68	12	126	18	18	17	53	High
70	46	66	11	120	16	17	16	51	
60	43	63	10	117	15	16	15	48	Average
50	41	61	9	111	14	15	13	47	
40	38	59	8	107	13	14	12	45	
30	35	56	7	99	12	13	11	43	Low
20	32	52	6	93	10	12	10	41	
10	25	48	5	85	8	11	9	37	Extremely low
1	10	38	2	70	4	6	4	29	

PC, Percentile; CC, Compassionate Care; PA, Perspective Adoption; WIPS, Walking in the Patient’s Shoes; EMP, Empathy; ENG, Engineering; ECO, Ecological; ADA, Adaptation.

The structural model of resilience as a predictor of empathy performed adequately, as evidenced by the fit indices (X2 = 654.77, df = 449, *p* < 0.001, CFI = 0.94, TLI = 0.93, RMSEA [90% CI] = 0.04 [0.03–0.04], SRMR = 0.05).

**Figure 2:**
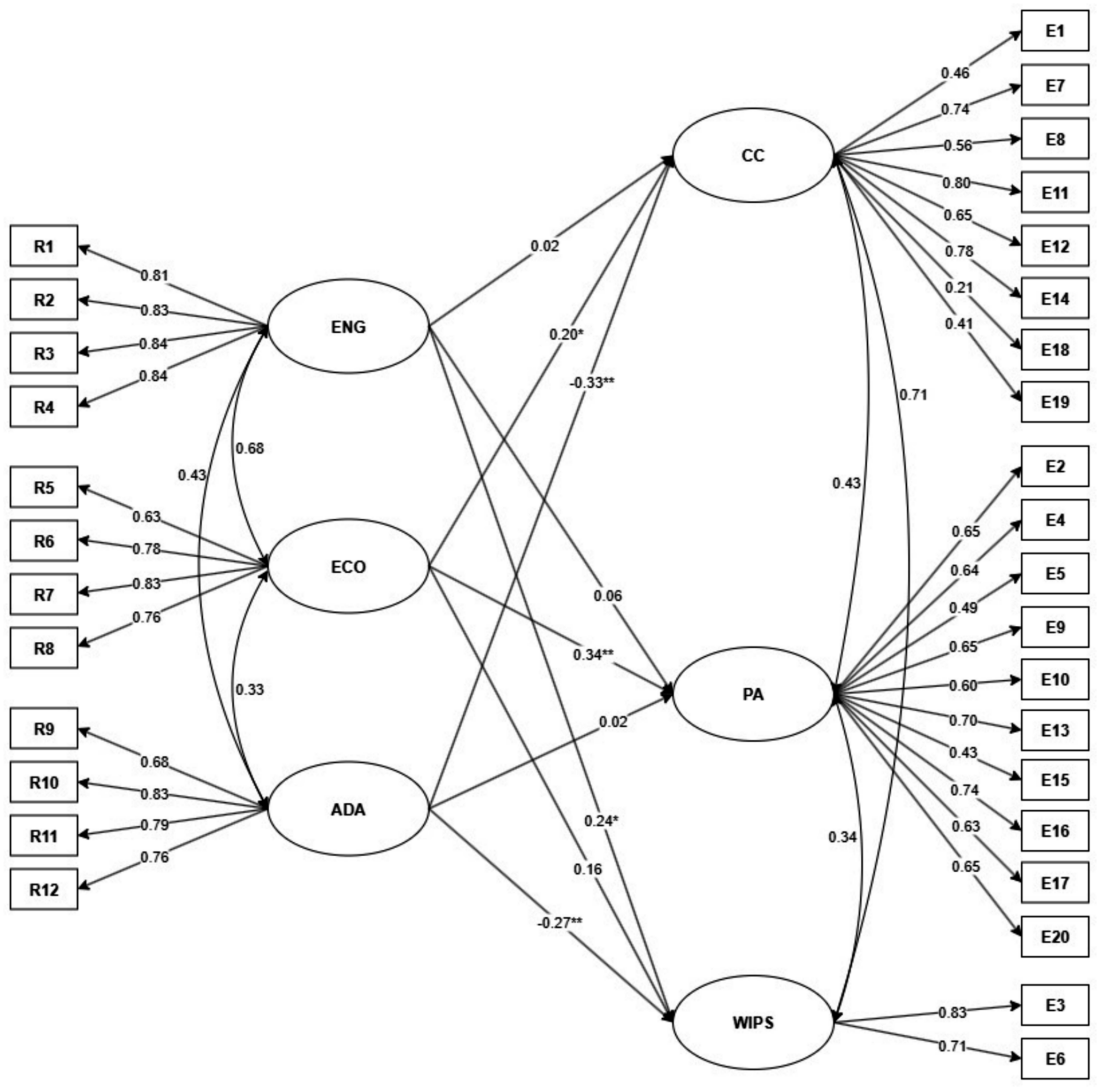
Structural model of resilience as a predictor of empathy. ENG, Engineering; ECO, Ecological; ADA, Adaptation; CC, Compassionate Care; PA, Perspective Adoption; WIPS, Walking in the Patient’s Shoes. **p < 0.01, *p < 0.05.

Figure 2 shows that the “engineering” dimension of resilience positively and significantly predicts the “compassionate care” dimension of empathy. The “ecological” dimension positively and significantly predicts the “compassionate care” and “perspective adoption” dimensions. The “adaptation” dimension negatively and significantly predicts the “compassionate care” and “walking in the patient’s shoes” dimensions.

## 4 Discussion

The fulfillment of the factorial (multidimensional) structure of any attribute is an indispensable condition for the correct application of structural equations (Ruiz et al., 2010), when exploring the relationships that may exist between two or more attributes. For this reason, some authors suggest the methodological need for a psychometric analysis of the data to verify the behavior of certain parameters that guarantee the application of structural equations. This procedure can avoid biases attributed to the failure to meet the factorial model and the original distribution of the items in each of the dimensions in accordance with the theoretical model (Ortiz and Fernández-Pera, 2018).

The skewness and kurtosis results indicated that some items deviate from a normal distribution. However, this does not impact the results, as MLR estimators were used to mitigate biases when normality is only slightly or moderately violated.

The CFA results confirm that the model, which includes three underlying dimensions for both attributes, fits the data well, ensuring that any potential model-related biases have been minimized.

The study found invariance across sexes for empathy but not for resilience. Consequently, comparisons of empathy dimensions between sexes are appropriate, whereas such comparisons cannot be made for resilience. Although conducting these comparisons is not an objective of the present study, it is important to note that research in Latin America has reported substantial sex-related variability in empathy levels, including differences across its dimensions (Díaz-Narváez et al., 2021). In contrast, no studies in this region have examined potential sex differences in resilience. Evidence from other regions suggests that women may exhibit lower resilience than men (Pan et al., 2024), but this issue remains unresolved and warrants further investigation.

Table 5 presents cutoff points for the empathy and resilience scales based on percentile distributions, using five descriptive classification levels. These categories are intended to provide a norm-referenced interpretation of scores within the sample, facilitating their descriptive and comparative use. However, they should not be interpreted as clinical thresholds or indicators of functional impairment, and their practical implications should be considered with caution.

Resilience and Empathy. The theoretical basis for considering resilience as an independent variable lies in its protective function (Masten et al., 2021). This function enables individuals to buffer the impact of adverse events and, in turn, to preserve and express the empathy developed through lived experience during the ontogenetic process of becoming a physician (Díaz-Narváez et al., 2025a; Díaz-Narváez et al., 2025b; Ulloque et al., 2023). The notion that resilience protects empathy is supported by evidence indicating that resilience has a biological substrate in the left orbitofrontal cortex, while the biological basis of empathy—particularly its cognitive component—is also linked to the orbitofrontal system (Huang et al., 2025; Kong et al., 2018). Within empathy, the cognitive component modulates the emotional component. Moreover, stress, anxiety, and depression—often arising from external disturbances—can be partially explained by their disruptive effect on normal neural functioning, leading to a reduced regulatory capacity of the orbitofrontal system over the limbic system. Accordingly, resilience may inhibit or mitigate the impact of such disturbances on empathic stability by preventing declines in the cognitive regulation of emotional responses. Nevertheless, the precise mechanism underlying the relationship between resilience and empathy remains a subject of ongoing debate.

Ecological Resilience. The primary function of ecological resilience is the capacity to withstand negative events (Díaz-Narváez et al., 2025b; Adler et al., 2021). The results of the prediction of the relationship between ecological resilience and each of the dimensions of empathy are positive. This result can be explained by the presence of specific resilience traits in the physicians studied for this dimension. Namely: conscientious personality, goal management, and characterized by their persistence in maintaining their psychological stability, resistance to disturbance, constant vigilance, and reorganization of their psychological structure, including mental processes that determine behaviors to adapt to and resist the negative event (Acosta-Martínez et al., 2024; Díaz-Narváez et al., 2025a; Díaz-Narváez et al., 2025b; Maltby et al., 2015; Ulloque et al., 2023).

When the observed values of ecological resilience (Table 4) are compared with the established cutoff points (Table 5), ecological resilience is classified as high. In contrast, physicians’ empathy levels are average for Compassionate Care (CC) and Perspective Adoption (PA), high for Walking in Patient’s Shoes (WIPS), and average for overall empathy. One possible explanation is that traits associated with ecological resilience enable individuals to effectively withstand adverse events, thereby allowing these empathy dimensions to be expressed at the levels indicated by the cutoff points. The implications of these findings for the specific empathic attitudes adopted by physicians in their interactions with patients fall beyond the scope of the present study.

Consequently, ecological resilience may function as a buffer against turbulence, preserving the physician’s intellectual and rational understanding of the patient’s condition and experiences through perspective adoption (PA), as well as the ability to comprehend the subjectivity of the patient’s thoughts while maintaining professional objectivity (WIPS). It also helps protect the capacity to share emotional states with the patient, as reflected in compassionate care (CC). The empathic capacities safeguarded by the traits associated with this dimension of resilience enable physicians to withstand adverse events and maintain these core components of empathy. Nevertheless, empathic expression may ultimately be constrained by the level of empathy attained during the physicians’ ontogenetic development, suggesting that resilience protects—but does not expand—empathic capacity beyond this developmental level.

Engineering Resilience. Engineering resilience serves to restore equilibrium following disruption. When resilience functions effectively, any resulting disequilibrium is mild; however, successful resistance to adverse events does not imply complete immunity to turbulence (Díaz-Narváez et al., 2025b). The traits that facilitate the reestablishment of equilibrium are typically linked to a personality marked by emotional stability—such as self-esteem, self-control, frustration tolerance, healthy emotion regulation, and self-confidence—which together predicts both the capacity for recovery and the speed at which it occurs (Díaz-Narváez et al., 2025a, Ulloque et al., 2023). In contrast, traits including anger, hostility, depression, rumination, excessive worry (Maltby et al., 2015), and negative emotions more broadly (Mader et al., 2023) hinder the recovery process.

In the present study, findings for engineering resilience parallel those observed for ecological resilience: physicians exhibit high levels of engineering resilience alongside average levels of empathy. Thus, the engineering resilience observed appears to support recovery from disruption while sustaining empathic expression at the level attained during physicians’ professional development—namely, an average level.

Adaptive Resilience. Adaptive resilience refers to an individual’s capacity—in this case, that of the physicians studied—to adjust, respond flexibly, innovate, and modify behavior to cope effectively with the ongoing presence of adverse events (Maltby et al., 2015; VanMeter, 2020). This adaptive process should be understood as active and continuous, involving sustained efforts to neutralize turbulence rather than passive resignation.

The findings indicate a negative predictive relationship between adaptive resilience and the Compassionate Care (CC) and Walking in Patient’s Shoes (WIPS) dimensions, alongside a positive relationship with the Perspective Adoption (PA) dimension. One possible explanation is a relative deficit in traits that support effective adaptation, such as cognitive and emotional flexibility, openness to social support, hopeful thinking, emotional awareness, and self-reflection. The continued presence of turbulence may therefore reflect a diminished capacity for emotional sharing, which could account for the observed reductions in CC and WIPS.

In conclusion, the physicians assessed are generally capable of withstanding and restoring the stability of their original psychological state. However, if the negative event continues, it may impact the Compassionate Care (CC) and Walking in the Patient’s Shoes (WIPS) dimensions. This effect suggests that certain traits linked to adaptability may be lacking, which could hinder the ability to express compassion and to understand the patient’s emotional state rationally.

By examining how each dimension of resilience interacts with the dimensions of empathy, we gained insight into how the traits within each dimension of empathy serve as protective factors. This approach allows us to identify which traits, either present or lacking in the evaluated physicians, may facilitate or inhibit the expression of empathy, ultimately affecting the intersubjective relationship that empathy can foster.

It is important to highlight that the inferences observed in this study were made in physicians working in public hospitals, and it is known that these types of hospitals are subjected to constant and high workload pressure (Gupta et al., 2024; Munabi-Babigumira et al., 2017; Padilla Rojas et al., 2019; Ramos-Zúñiga et al., 2018). The results of similar studies conducted in different populations of healthcare professionals (Acosta-Martínez et al., 2024; Díaz-Narváez et al., 2025a; Díaz-Narváez et al., 2025b; Ulloque et al., 2023), show variability in the form (positive or negative relationship) and magnitude (*β* values) of their results. This variability may be due to the fact that the same negative event can present itself in different magnitudes and forms. At the same time, these same events, in themselves, can be variable (of different origins) (Cao et al., 2024; Chien et al., 2024; Teo et al., 2024; Riess, 2021). Consequently, this finding should be studied to find well-founded working hypotheses that guide a better explanation of this variability. On the other hand, it is known that empathy can be influenced by several factors that, in turn, can act with different intensities (Li et al., 2024; Maximiano-Barreto et al., 2020; Rajput et al., 2020). These circumstances may explain the low values of the β coefficients found in the present study, regardless of whether the results are statistically significant or not.

## 5 Limitations

The main limitation of this study is that the sample is not representative of the studied population. The findings may only be valid for the studied population in terms of trends.

The sample included both medical specialists and residents, who were analyzed as a single group due to shared clinical functions and sample size considerations. Future studies with balanced subgroup samples should examine potential differences in empathy and resilience across levels of professional training.

## Data Availability

The database used in this study is available in the OSF repository: https://osf.io/axgw2/overview?view_only=2b80b29131b84cac91c1a974c2a3c1fa.
